# Supplementary material for: Targeted randomization dose optimization trials enable fractional dosing of scarce drugs
Source: PLoS One. 2023 Oct 30;18(10):e0287511. doi: 10.1371/journal.pone.0287511 (PMC10615276; doi:10.1371/journal.pone.0287511)
Supplement: S1 Appendix — (DOCX) [file pone.0287511.s001.docx]

**Supplemental Appendix**

**Title:** Targeted randomization dose optimization trials enable fractional dosing of scarce drugs

**Running Head:** Dose optimization of scarce medical resources

**Authors:** Philip S. Boonstra, PhD^1,2^, Alex Tabarrok, PhD^3^, and Garth W Strohbehn, MD, MPhil^2,4,5^

**Affiliations:**

1. Department of Biostatistics, University of Michigan, Ann Arbor, MI, USA
2. Rogel Cancer Center, University of Michigan, Ann Arbor, MI, USA
3. Department of Economics, George Mason University, Fairfax, VA, USA
4. Veterans Affairs Center for Clinical Management and Research, Ann Arbor, MI, USA
5. Division of Medical Oncology, VA Ann Arbor, Ann Arbor, MI, USA

**Journal:** *PLOS One*

**Article Type:** Article

**Classification:** Medical sciences, statistics, economic sciences

**Key words:** operating characteristics, vaccines, dose stretching, fractional dosing, pandemic, dose-response

**Supplemental Figures/Tables:** 18 supplemental tables

**Funding:** PSB and GWS are supported in this work by P30CA046592.

**Conflict of Interest**: PSB has received research funding from Bristol Myers Squibb and Janssen outside of the submitted work. AT has no conflicts to disclose. GWS serves as an uncompensated Director of the Optimal Cancer Care Alliance. GWS is a co-inventor of a patent held by the University of Chicago covering the use of low-dose tocilizumab in the treatment of viral infections. GWS reports no material conflicts of interest with regards to contract research organizations, biostatistical firms, or vaccines.

**Disclosures:** GWS is employed by the United States Department of Veterans Affairs; this work does not represent the official position of the United States Department of Veterans Affairs.

**Contributions:** PSB and GWS designed research, performed research, interpreted research, and wrote initial drafts of the manuscript. AT interpreted research and provided intellectually important contributions to drafts of the manuscript. All authors have reviewed and approve of the final version of the manuscript.

**Contact Information:**

Garth W Strohbehn, MD, MPhil

Veterans Affairs Center for Clinical Management and Research

University of Michigan North Campus Research Complex

Building 16, Office 322W

Ann Arbor, MI, USA 48109

Email: gstrohbe@umich.edu

**Supplemental Methods**

**Isotonic Probability Vector Distributions**

The horseshoe isotonic probability vector distribution [24] on the non-negative weights $\alpha_{1}, \alpha_{2}, \ldots, \alpha_{T+1}$ (which share a 1-1 correspondence with the response parameters $\xi_{1},\xi_{2},\ldots,\xi_{T}$) is characterized by the following distributions:

$$\alpha_{j}|\tau,\lambda_{j}\sim N^{+}\left( 0,\frac{c^{2}\tau^{2}\lambda_{j}^{2}}{1+c^{2}\tau^{2}\lambda_{j}^{2}} \right), \lambda_{j}\sim C^{+}(0,1), \tau\sim C^{+}(0,1)$$

The N^+^(a,b^2^) notation indicates the normal distribution with mean a and variance b^2^ with support truncated to the positive half of the real line, and C^+^(a,b^2^) indicates the Cauchy distribution with location a and scale b with support truncated to the positive half of the real line. In words, this means that each $\alpha_{j}$ is a priori conditionally distributed as a half-normal random variable given $\lambda_{j}$ and $\tau$, each $\lambda_{j}$ is a priori distributed as a half-Cauchy random variable, and each $\tau$ is a priori distributed as a half-Cauchy random variable. In the present context, the value of $\alpha_{j}$, j = 2, …, T, corresponds to the relative increase in response probability between dose level j-1 and dose level j; a larger value implies a larger increase in probability. The $\lambda_{j}$s and $\tau$ are hyperparameters, referred to as ‘local’ and ‘global’ shrinkage parameters, respectively.

These prior distributions are updated in the usual Bayesian way as data from the trial are collected (see below for an efficient implementation of this update). For the Naïve, Greedy, and TR approaches, updates are required after each individual subject in the trial. Thus, the posterior distribution of the $\alpha_{j}$s will vary throughout the trial. For TRCoh, the distributions are updated after 1/3 and 2/3 of the planned sample size and again when the trial is complete. When an update is made, if the data suggest that there is a relatively large jump in dose-response probability between two consecutive dose levels, then the corresponding value of $\lambda_{j}$ at the higher dose level will be large, which increases the variance of the $\alpha_{j}$ in the half-normal distribution.

The horseshoe-plus prior incorporates an additional hyperparameter into the HSIPV distribution:

$$\alpha_{j}|\tau,\lambda_{j}, \gamma_{j}\sim N^{+}\left( 0,\frac{c^{2}\tau^{2}\lambda_{j}^{2}\gamma_{j}^{2}}{1+c^{2}\tau^{2}\gamma_{j}^{2}} \right), \gamma_{j}\sim C^{+}(0,1),\lambda_{j}\sim C^{+}(0,1), \tau\sim C^{+}(0,1)$$

This additional hyperparameter $\gamma_{j}$ is essentially a second local shrinkage parameter, thus allowing for even sharper distinctions between dose levels. Readers should consult references [24-30] from the manuscript for further information.

**Computationally efficient Bayesian update**

Bayesian updates are generally non-trivial and computationally intensive, a problem which is exacerbated in the present context, since the model potentially needs to be updated after every single subject in the trial. However, we can leverage the fact that the dimensionality of the parameters of interest ($\boldsymbol{\xi}=\xi_{1},\xi_{2},\ldots,\xi_{T}$) is small to conduct an efficient Bayesian update. Specifically, Bayes theorem gives that the posterior distribution of $\boldsymbol{\xi}$ given the data from the first n subjects, $\boldsymbol{D}_{n}$, is proportional to the likelihood of the data times the prior, or

$$\pi\left( \boldsymbol{\xi} | \boldsymbol{D}_{n} \right)\boldsymbol{\propto}L\left( \boldsymbol{D}_{n} | \boldsymbol{\xi} \right)\pi\left( \boldsymbol{\xi} \right)$$

Thus, we can obtain draws from the *posterior* distribution of $\boldsymbol{\xi}$ by resampling draws from its *prior* distribution with importance resampling weights proportional to the likelihood of the data at each prior draw. The likelihood calculation is very fast, and the prior only needs to be sampled once at the beginning of the trial. Whenever the posterior needs to be updated, e.g. after the data are available from a new subject and a new dose assignment is needed, we only need to recalculate the importance resampling weights and then resample the draws. A similar relationship can be exploited to calculate the posterior probability that each dose is the MDSE:

$$\rho_{t}\left( n \right)\equiv\Pr\left( \xi_{t-1}\boldsymbol{<}k\xi_{T}\boldsymbol{\leq}\xi_{t} | \boldsymbol{D}_{n} \right)\boldsymbol{=}E\left( 1_{\left[ \xi_{t-1}\boldsymbol{<}k\xi_{T}\boldsymbol{\leq}\xi_{t} \right]} | \boldsymbol{D}_{n} \right)$$

$$= \int1_{\left[ \xi_{t-1}\boldsymbol{<}k\xi_{T}\boldsymbol{\leq}\xi_{t} \right]} \pi\left( \boldsymbol{\xi} | \boldsymbol{D}_{n} \right)d\boldsymbol{\xi}$$

$$= \int1_{\left[ \xi_{t-1}\boldsymbol{<}k\xi_{T}\boldsymbol{\leq}\xi_{t} \right]} \frac{L\left( \boldsymbol{D}_{n} | \boldsymbol{\xi} \right)\pi\left( \boldsymbol{\xi} \right)}{L\left( \boldsymbol{D}_{n} \right)}d\boldsymbol{\xi}$$

$$= \frac{\int1_{\left[ \xi_{t-1}\boldsymbol{<}k\xi_{T}\boldsymbol{\leq}\xi_{t} \right]} L\left( \boldsymbol{D}_{n} | \boldsymbol{\xi} \right)\pi\left( \boldsymbol{\xi} \right)d\boldsymbol{\xi}}{\int L\left( \boldsymbol{D}_{n} | \boldsymbol{\xi} \right)\pi\left( \boldsymbol{\xi} \right)d\boldsymbol{\xi}}$$

$$\approx\frac{\sum_{\tilde{\boldsymbol{\xi}}\sim\pi\left( \boldsymbol{\xi} \right)} 1_{\left[ \xi_{t-1}\boldsymbol{<}k\xi_{T}\boldsymbol{\leq}\xi_{t} \right]} L\left( \boldsymbol{D}_{n} | \boldsymbol{\xi} \right)}{\sum_{\tilde{\boldsymbol{\xi}}\sim\pi\left( \boldsymbol{\xi} \right)} L\left( \boldsymbol{D}_{n} | \boldsymbol{\xi} \right)}$$

The notation 1_x_ is the indicator function, equal to 1 if the argument x is true, and 0 otherwise. In our simulation study, we sampled 150,000 draws from the prior $\pi\left( \boldsymbol{\xi} \right)$**,** and, whenever a Bayesian update was required, resampled 75,000 draws with replacement and with importance resampling weights proportional to the likelihood at the current data configuration.

**Supplemental Results**

**Societal Benefit: True MDSE Identification**

*Sensitivity of True MDSE Identification to Sample Size*

Simulations were repeated with trial sample size of N=144 and N=201; results are summarized in **Supplemental Table 3**. Increasing sample size from 102 to 201 failed to reliably improve MDSE identification by the Naïve or Gr dose allocation strategies. As expected, increasing sample size from 102 to 201 improved MDSE identification by EA (e.g., for curve 5, N=102 accuracy 64% vs N=144 accuracy 73% vs N=201 accuracy 78%). Increasing sample size from 102 to 201 improved MDSE identification by both TRCoh and TRInd, though the magnitude of improvement was greater for TRCoh than TRInd and for both TR strategies was less than the magnitude of improvement in MDSE identification of EA.

*Sensitivity of True MDSE Identification to Upper Asymptote (Absolute Efficacy)*

As expected, decreasing the hypothetical drug’s maximum absolute efficacy reduced MDSE identification performance across all dose-response curves, sample sizes, prior distributions, and dose allocation strategies; results are summarized in **Supplemental Table 4**. For curves where EA’s advantage over TR was most pronounced at high absolute efficacy (e.g., curves 1 and 2), the decline in MDSE identification was stark (e.g., for EA, curve 2 accuracy 41% vs curve 10 accuracy 23% vs curve 18 accuracy 14% vs curve 26 accuracy 5%), reflecting ~88% relative reduction in MDSE identification rate with 52% reduction in maximal efficacy. Similarly, TRCoh and TRInd had ~92% and ~86% relative reductions in MDSE identification rates, respectively. Of note, in the set comprised of a given dose allocation strategy (other than Gr) and *analogous* dose-response curves (e.g., curves 1, 9, 17, 25), *decreasing* maximum absolute efficacy led to a reduction in *absolute* performance. Within the set comprised of a given dose allocation strategy (other than Gr) and set of dose-response curves with equal maximum absolute efficacy (e.g., curves 1-8), *relative* performance was largely preserved. Dose-response curves with the lowest *true* MDSE curves were disproportionately impacted by reductions in maximum absolute efficacy.

*Sensitivity of True MDSE Identification to Prior Distribution*

We evaluated both the HS and HSPL prior distributions; results are summarized in **Supplemental Table 5**. Across dose allocation strategies and a given dose-response curve, there was a pattern to the relative performances of HS and HSPL: HSPL reliably outperformed HS for dose-response curves 1-3 (as well as 9-11; 17-19; and 25-27) and HS reliably outperformed HSPL for dose-response curves 6-8 (as well as 14-16; 22-24; and 30-32). Among the dose-response curves for which true MDSE was at a lower dose level (e.g., curves 1, 2, and 3), HSPL offered comparatively better protection against decreases in MDSE identification rate arising from declines in maximum absolute efficacy. For example, with TRCoh, from curve 1 to curve 9 to curve 17 to curve 25, HSPL had ~74% relative reduction in true MDSE identification rate, as compared to ~92% for HS. By comparison, HS did not offer the same degree of protection: Again, with TRCoh, from curve 6 to curve 14 to curve 22 to curve 30, HSPL had 30% relative decrease (17% absolute decrease) in true MDSE identification rate, compared to 32% relative decrease (22% absolute decrease) for HS. The magnitude of HSPL’s detection advantage over HS for curves 1-3 (and qualitatively analogous dose-response curves) was greater than the magnitude of HS’s detection advantage over HSPL for curves 6-8 (and qualitatively analogous dose-response curves).

**Societal Benefit: Probability of** **Individual Assignment to True MDSE as Proxy for Speed**

*Sensitivity of Individual Assignment to True MDSE to Sample Size*

Simulations were repeated with trial sample size of n=144 and n=201; results are summarized in **Supplemental Table 6**. Increasing sample size from 102 to 201 had minimal impact on the likelihood of an individual clinical trial participant receiving the true MDSE, particularly for EA, Naïve, and Gr. TRInd and TRCoh demonstrated small increases in the likelihood of assignment to true MDSE as sample size increased (e.g., for curve 7 for TRCoh, n=102, 61% vs n=144, 67% vs n=201, 70%).

*Sensitivity of Individual Assignment to True MDSE to Upper Asymptote (Absolute Efficacy)*

Decreasing the hypothetical drug’s maximum absolute efficacy consistently reduced the likelihood of assignment to true MDSE for all model-based (i.e., non-EA) designs (**Supplemental Table 7**). Compared to EA, TRInd and TRCoh lost their MDSE assignment advantage for curves for which 1) MDSE was a very low dose and 2) absolute efficacy was low. For example, TRInd had MDSE assignment probabilities of 28% for curve 2, 18% for curve 10, 11% for curve 18, and 6% for curve 26, compared to EA’s 17%. When fewer dose de-escalation steps were required, TR strategies retained significant MDSE assignment advantage over EA. TRInd had generally higher probability of MDSE assignment than TRCoh for a given dose-response curve, consistent with updated probability assessments after each outcome.

*Sensitivity of Individual Assignment to True MDSE to Prior Distribution*

We evaluated both the HS and HSPL prior distributions; results are summarized in **Supplemental Table 8**. Across dose allocation strategies and a given dose-response curve, there was a pattern to the relative performances of HS and HSPL in terms of MDSE assignment: HSPL reliably outperformed HS for dose-response curves 1-3 and HS tended to outperform HSPL at other dose-response curves, consistent with the frequency with which the outcome of interest (response) occurred. The magnitude of HSPL’s detection advantage over HS for curves 1-3 (and qualitatively analogous dose-response curves), which was a matter of a few percentage points of MDSE assignment probability, was approximately the same magnitude as HS’s MDSE assignment advantage over HSPL for other curves.

**Societal Risk: De-Escalation from *T* with MDSE Underestimation**

*Sensitivity of MDSE Underestimation to Sample Size*

No appreciable changes in MDSE underestimation rate were noted with increasing sample size. For example, in EA increasing sample size from n=102 to n=144 led to *increases* in MDSE underestimation rate in true dose-response curve 6 and minor *decreases* in MDSE underestimation rate in true dose-response curve 3. Results are summarized in **Supplemental Table 9**.

*Sensitivity of MDSE Underestimation to Upper Asymptote (Absolute Efficacy)*

Decreasing absolute efficacy was largely associated with an increased risk of MDSE underestimation (**Supplemental Table 10**). The largest increase in risk of MDSE underestimation was appreciated with the transition from upper asymptote of 0.84 (curves 1-8) to 0.7 (curves 9-16): Across all analogous true dose-response curves, MDSE underestimation risk increased in EA, Naïve, TRCoh, and TRInd. Curiously, risk of MDSE underestimation with Gr *decreased* as absolute efficacy decreased. Curious, idiosyncratic differences between EA and TRCoh emerged in certain analogous true dose-response curves. For example, across the set of true dose-response curves 6, 14, 22, and 30, the risk of MDSE underestimation *decreased* for EA (from 37% to 28%) while the risk of MDSE underestimation *increased* for TRCoh (from 27% to 35%).

*Sensitivity of MDSE Underestimation to Prior Distribution*

We evaluated the effect of the HS and HSPL prior distributions on MDSE underestimation risk; results are summarized in **Supplemental Table 11**. Across dose allocation strategies and within a given dose-response curve, the risk of MDSE underestimation was considerably higher with the HSPL prior distribution. The magnitude of the difference between HS and HSPL was greatest for those true dose-response curves in which the dose level immediately lower than true MDSE was close to the relative efficacy threshold (e.g., Curves 4 and 6). The magnitude of the difference between HS and HSPL was greatest in the TRCoh and TRInd allocation schema.

**Societal Risk: Variability of Efficacy Estimates as Assessed by Root-Mean-Squared Error (RMSE)**

*Sensitivity of RMSE of Estimated MDSE Efficacy to Sample Size*

Across all allocation schema and true dose-response curves, RMSE decreased with increasing sample size. Similar trend was observed across true dose-response curves of all relative efficacies – Regardless of the estimated maximal efficacy of the true dose-response curve, increasing sample size reduced RMSE. Similarly, sample size’s ability to improve RMSE was independent of the prior distribution employed (**Supplemental Table 13**).

*Sensitivity of RMSE of Estimated MDSE Efficacy to Upper Asymptote (Absolute Efficacy)*

We assessed the accuracy of MDSE efficacy estimates across the true dose-response curves (**Supplemental Table 14**). As maximum efficacy decreased, RMSE responses were idiosyncratic and dose allocation schema-dependent. For example, RMSE of efficacy estimates increased in simulated trials employing EA as the upper asymptote of efficacy decreased (e.g., 0.071 for curve 6 versus 0.081 for curve 30). Similarly, RMSE increased more dramatically using Gr allocation (e.g., 0.044 for curve 6 versus 0.139 for curve 30). RMSE increases were less pronounced for Naïve allocation (e.g., 0.038 for curve 6 versus 0.048 for curve 30). Targeted randomization allocation schemas were relatively resistant to RMSE changes as the upper asymptote of efficacy decreased: TRInd, for example, saw an increase from 0.051 for curve 6 to 0.057 for curve 30, while TRCoh RMSE remained stable (0.057 for both curve 6 and curve 30).

*Sensitivity of RMSE of Estimated MDSE Efficacy to Prior Distribution*

We evaluated the effect of the HS and HSPL prior distributions on RMSE of estimated efficacy; results are summarized in **Supplemental Table 15**. Across dose allocation strategies and within a given dose-response curve, RMSE of estimated efficacy was higher with the HSPL prior distribution. The magnitude of the difference between HS and HSPL was small, however, with absolute differences most commonly less than 0.01, reflecting excellent accuracy in RMSE estimation. Of note, the magnitude of difference between HS and HSPL was particularly notable for Gr dose allocation when the upper asymptote of efficacy decreased.

**Individual Risk: Probability of Underdosing**

*Sensitivity of Probability of Underdosing to Sample Size*

Simulations were repeated with trial sample size of N=144 and N=201; results are summarized in **Supplemental Table 16**. For a given true dose-response curve, increasing sample size from 102 to 201 offered minor benefit in preventing sub-true MDSE dosing. Underdosing in EA was not impacted by changes in sample size. Underdosing of the model-based allocation strategies, relative to one another, did not meaningfully change with increases in sample size; in order of ascending probability of underdosing, Naïve < TRInd < TRCoh < Gr, for nearly any given curve. Increasing sample size had comparatively little impact on the rate of underdosing in TRCoh for all except curves 7 and 8 (e.g., curve 3 underdosing rate of 10% with n=102 vs 10% with n=144 vs 10% with n=201).

*Sensitivity of Probability of Underdosing to Upper Asymptote (Absolute Efficacy)*

We assessed the effect of upper asymptote on the likelihood of an individual subject receiving a dose less than true MDSE in the simulated clinical trials. (**Supplemental Table 17**) Probability of sub-true MDSE dosing was consistent for EA across all levels of maximal absolute efficacy. While the probability of sub-true MDSE dosing using Gr *decreased* as maximal absolute efficacy decreased (e.g., 41% for curve 6 versus 25% for curve 30), the probability *increased* using Naïve allocation (e.g., 10% for curve 8 versus 20% for curve 32). Among Targeted Randomization allocation schema, changes to the probability of sub-true MDSE dosing with decreasing maximal absolute efficacy varied depending on the true dose-response curve. For example, probability of sub-true MDSE dosing using TRCoh increased for analogous curves 7, 15, 23, and 31 (20% to 24% to 29% to 35%) and decreased for analogous curves 4, 12, 20, and 28 (27% to 21% to 17% to 14%). Similar trends, albeit more muted, were appreciated with TRInd.

*Sensitivity of Probability of Underdosing to Prior Distribution*

We evaluated the effect of the HS and HSPL prior distributions on sub-true MDSE dosing; results are summarized in **Supplemental Table 18**. Across dose allocation strategies and within a given dose-response curve, the risk of sub-true MDSE dosing was consistent 2-4% higher using the HSPL prior distribution. The magnitude of the difference between HS and HSPL was greatest in the TRInd allocation schema.

**Supplemental Table 1. Hill equation parameters of true dose-response curves in simulated clinical trials.** Summarized parameters of the true dose-response curves visualized in Figure 1. Four-parameter Hill equation is: Pr(R=1|X) = a+ (b-a)/([1+(c/X)^d]). Abbreviations: EC_50_, concentration achieving half of the maximum possible increase in probability of efficacy.

| **Curve Number** | **b**  **(Upper Asymptote, Efficacy)** | **d**  **(Hill slope)** | **a**  **(Efficacy of Dose Level 0)** | **c**  **(EC_50_)** |
| --- | --- | --- | --- | --- |
| Curve 1 | 0.85 | 1 | 0.20 | 0.70 |
| Curve 2 | 0.85 | 2 | 0.20 | 0.70 |
| Curve 3 | 0.85 | 3 | 0.20 | 0.70 |
| Curve 4 | 0.85 | 4 | 0.20 | 0.70 |
| Curve 5 | 0.85 | 5 | 0.20 | 0.70 |
| Curve 6 | 0.85 | 7 | 0.20 | 0.70 |
| Curve 7 | 0.85 | 10 | 0.20 | 0.70 |
| Curve 8 | 0.85 | 20 | 0.20 | 0.70 |
| Curve 9 | 0.70 | 1 | 0.20 | 0.70 |
| Curve 10 | 0.70 | 2 | 0.20 | 0.70 |
| Curve 11 | 0.70 | 3 | 0.20 | 0.70 |
| Curve 12 | 0.70 | 4 | 0.20 | 0.70 |
| Curve 13 | 0.70 | 5 | 0.20 | 0.70 |
| Curve 14 | 0.70 | 7 | 0.20 | 0.70 |
| Curve 15 | 0.70 | 10 | 0.20 | 0.70 |
| Curve 16 | 0.70 | 20 | 0.20 | 0.70 |
| Curve 17 | 0.55 | 1 | 0.20 | 0.70 |
| Curve 18 | 0.55 | 2 | 0.20 | 0.70 |
| Curve 19 | 0.55 | 3 | 0.20 | 0.70 |
| Curve 20 | 0.55 | 4 | 0.20 | 0.70 |
| Curve 21 | 0.55 | 5 | 0.20 | 0.70 |
| Curve 22 | 0.55 | 7 | 0.20 | 0.70 |
| Curve 23 | 0.55 | 10 | 0.20 | 0.70 |
| Curve 24 | 0.55 | 20 | 0.20 | 0.70 |
| Curve 25 | 0.40 | 1 | 0.20 | 0.70 |
| Curve 26 | 0.40 | 2 | 0.20 | 0.70 |
| Curve 27 | 0.40 | 3 | 0.20 | 0.70 |
| Curve 28 | 0.40 | 4 | 0.20 | 0.70 |
| Curve 29 | 0.40 | 5 | 0.20 | 0.70 |
| Curve 30 | 0.40 | 7 | 0.20 | 0.70 |
| Curve 31 | 0.40 | 10 | 0.20 | 0.70 |
| Curve 32 | 0.40 | 20 | 0.20 | 0.70 |

**Supplemental Table 2. Sensitivity of true MDSE identification to changes in sample size.** Cells contain the percentage of simulated trials that identified the true MDSE for a given combination of dose allocation strategy and dose-response curve across N=500 simulated trials, with sample size ranging from n=102 to n=144 to n=201. Proportions are derived from N=500 simulated trials, using HS prior distribution and Hill equation parameters corresponding to those in **Supplemental Table 1**. Darker green color represents a higher probability of this ‘good’ event; darker red represents a lower probability of this ‘good’ event. **Abbreviations:** EA, equal allocation; Naïve, naïve allocation; Gr, Greedy allocation; TRInd, targeted randomization (individual); TRCoh, targeted randomization (cohort, comprising 1/3 of study population).

| **Sample Size** | **Allocation Schema** | **Curve 1** | **Curve 2** | **Curve 3** | **Curve 4** | **Curve 5** | **Curve 6** | **Curve 7** | **Curve 8** |
| --- | --- | --- | --- | --- | --- | --- | --- | --- | --- |
|  | **EA** | 85% | 41% | 53% | 56% | 64% | 56% | 84% | 84% |
|  | **Naïve** | 0% | 0% | 0% | 0% | 0% | 65% | 58% | 94% |
| **102** | **Gr** | 49% | 7% | 26% | 70% | 55% | 77% | 87% | 81% |
|  | **TRInd** | 75% | 22% | 49% | 73% | 69% | 73% | 94% | 90% |
|  | **TRCoh*** | 78% | 39% | 59% | 66% | 72% | 69% | 92% | 93% |
|  |  |  |  |  |  |  |  |  |  |
|  | **EA** | 88% | 48% | 66% | 56% | 73% | 57% | 89% | 89% |
|  | **Naïve** | 0% | 0% | 0% | 0% | 0% | 66% | 59% | 96% |
| **144** | **Gr** | 54% | 6% | 30% | 71% | 56% | 80% | 90% | 86% |
|  | **TRInd** | 81% | 17% | 43% | 71% | 69% | 72% | 96% | 86% |
|  | **TRCoh*** | 80% | 40% | 60% | 69% | 79% | 69% | 93% | 97% |
|  |  |  |  |  |  |  |  |  |  |
|  | **EA** | 94% | 49% | 69% | 64% | 78% | 57% | 91% | 92% |
|  | **Naïve** | 0% | 0% | 0% | 0% | 0% | 66% | 58% | 95% |
| **201** | **Gr** | 51% | 5% | 25% | 76% | 56% | 79% | 93% | 84% |
|  | **TRInd** | 86% | 21% | 52% | 79% | 76% | 73% | 98% | 91% |
|  | **TRCoh*** | 89% | 44% | 67% | 76% | 82% | 74% | 97% | 99% |

**Supplemental Table 3. Sensitivity of true MDSE identification to changes in upper asymptote (estimated maximal efficacy).** Cells contain the percentage of simulated trials that identified the true MDSE for a given combination of dose allocation strategy and dose-response curve across N=500 simulated trials. Proportions are derived from N=500 simulated trials, using HS prior distribution, n=102, and Hill equation parameters corresponding to those in **Supplemental Table 1**. The upper asymptote of efficacy ranges from 0.85 (curves 1-8) to 0.40 (curves 25-32). Darker green color represents a higher probability of this ‘good’ event; darker red represents a lower probability of this ‘good’ event. **Abbreviations:** EA, equal allocation; Naïve, naïve allocation; Gr, Greedy allocation; TRInd, targeted randomization (individual); TRCoh, targeted randomization (cohort, comprising 1/3 of study population).

| **Allocation Schema** | **Curve 1** | **Curve 2** | **Curve 3** | **Curve 4** | **Curve 5** | **Curve 6** | **Curve 7** | **Curve 8** |
| --- | --- | --- | --- | --- | --- | --- | --- | --- |
| **EA** | 85% | 41% | 53% | 56% | 64% | 56% | 84% | 84% |
| **Naïve** | 0% | 0% | 0% | 0% | 0% | 65% | 58% | 94% |
| **Gr** | 49% | 7% | 26% | 70% | 55% | 77% | 87% | 81% |
| **TRInd** | 75% | 22% | 49% | 73% | 69% | 73% | 94% | 90% |
| **TRCoh*** | 78% | 39% | 59% | 66% | 72% | 69% | 92% | 93% |
|  | **Curve 9** | **Curve 10** | **Curve 11** | **Curve 12** | **Curve 13** | **Curve 14** | **Curve 15** | **Curve 16** |
| **EA** | 52% | 23% | 38% | 48% | 50% | 52% | 73% | 79% |
| **Naïve** | 0% | 0% | 0% | 0% | 0% | 55% | 49% | 92% |
| **Gr** | 5% | 1% | 7% | 31% | 26% | 48% | 48% | 88% |
| **TRInd** | 38% | 19% | 33% | 54% | 60% | 63% | 86% | 78% |
| **TRCoh*** | 39% | 19% | 40% | 58% | 58% | 59% | 83% | 87% |
|  | **Curve 17** | **Curve 18** | **Curve 19** | **Curve 20** | **Curve 21** | **Curve 22** | **Curve 23** | **Curve 24** |
| **EA** | 27% | 14% | 26% | 41% | 40% | 52% | 67% | 75% |
| **Naïve** | 0% | 0% | 0% | 0% | 0% | 44% | 42% | 48% |
| **Gr** | 2% | 0% | 1% | 10% | 4% | 42% | 38% | 98% |
| **TRInd** | 24% | 7% | 26% | 46% | 46% | 60% | 80% | 68% |
| **TRCoh*** | 18% | 9% | 23% | 47% | 50% | 51% | 79% | 75% |
|  | **Curve 25** | **Curve 26** | **Curve 27** | **Curve 28** | **Curve 29** | **Curve 30** | **Curve 31** | **Curve 32** |
| **EA** | 10% | 5% | 18% | 34% | 35% | 45% | 54% | 59% |
| **Naïve** | 0% | 0% | 0% | 0% | 0% | 37% | 38% | 82% |
| **Gr** | 0% | 0% | 0% | 1% | 1% | 8% | 5% | 100% |
| **TRInd** | 8% | 3% | 14% | 44% | 41% | 58% | 73% | 53% |
| **TRCoh*** | 6% | 3% | 15% | 45% | 43% | 47% | 65% | 61% |

**Supplemental Table 4. Sensitivity of true MDSE identification to changes in the prior distribution (HS or HSPL).** Cells contain the percentage of simulated trials that identified the true MDSE for a given combination of dose allocation strategy and dose-response curve across N=500 simulated trials. Proportions are derived from N=500 simulated trials, using n=102 and Hill equation parameters corresponding to those in **Supplemental Table 1**. Darker green color represents a higher probability of this ‘good’ event; darker red represents a lower probability of this ‘good’ event. **Abbreviations:** EA, equal allocation; Naïve, naïve allocation; Gr, Greedy allocation; TRInd, targeted randomization (individual); TRCoh, targeted randomization (cohort, comprising 1/3 of study population).

| **Prior Distribution** | **Allocation Schema** | **Curve 1** | **Curve 2** | **Curve 3** | **Curve 4** | **Curve 5** | **Curve 6** | **Curve 7** | **Curve 8** |
| --- | --- | --- | --- | --- | --- | --- | --- | --- | --- |
|  | **EA** | 85% | 41% | 53% | 56% | 64% | 56% | 84% | 84% |
|  | **Naïve** | 0% | 0% | 0% | 0% | 0% | 65% | 58% | 94% |
| **HS** | **Gr** | 49% | 7% | 26% | 70% | 55% | 77% | 87% | 81% |
|  | **TRInd** | 75% | 22% | 49% | 73% | 69% | 73% | 94% | 90% |
|  | **TRCoh*** | 78% | 39% | 59% | 66% | 72% | 69% | 92% | 93% |
|  |  |  |  |  |  |  |  |  |  |
|  | **EA** | 91% | 56% | 60% | 48% | 65% | 47% | 82% | 80% |
|  | **Naïve** | 0% | 0% | 0% | 0% | 0% | 62% | 42% | 90% |
| **HSPL** | **Gr** | 49% | 12% | 31% | 53% | 53% | 64% | 81% | 70% |
|  | **TRInd** | 85% | 33% | 50% | 58% | 68% | 59% | 92% | 84% |
|  | **TRCoh*** | 86% | 51% | 63% | 56% | 73% | 57% | 90% | 91% |

**Supplemental Table 5. Probability of individual assignment to true MDSE.** Cells contain the average percentage of subjects that were assigned to the true MDSE for a given combination of dose allocation strategy and dose-response curve across N=500 simulated trials. Proportions are derived from N=500 simulated trials, using HS prior distribution, sample size n=102, and Hill equation parameters corresponding to those in **Supplemental Table 1.** Darker green color represents a higher probability of this ‘good’ event; darker red represents a lower probability of this ‘good’ event. **Abbreviations**: EA, equal allocation; Naïve, Naïve allocation; Gr, Greedy allocation; TRInd, targeted randomization (individual); TRCoh, targeted randomization (cohort, comprising 1/3 of study population).

| **Allocation Schema** | **Curve 1** | **Curve 2** | **Curve 3** | **Curve 4** | **Curve 5** | **Curve 6** | **Curve 7** | **Curve 8** |
| --- | --- | --- | --- | --- | --- | --- | --- | --- |
| **EA** | 17% | 17% | 17% | 17% | 17% | 17% | 17% | 17% |
| **Naïve** | 0% | 0% | 0% | 0% | 0% | 63% | 56% | 90% |
| **Gr** | 46% | 18% | 39% | 65% | 67% | 56% | 83% | 36% |
| **TRInd** | 58% | 28% | 45% | 53% | 56% | 50% | 74% | 68% |
| **TRCoh*** | 20% | 16% | 25% | 42% | 47% | 39% | 61% | 59% |

**Supplemental Table 6. Sensitivity of probability of individual assignment to true MDSE to changes in sample size.** Cells contain the average percentage of subjects that were assigned to true MDSE for a given combination of dose allocation strategy and dose-response curve across N=500 simulated trials, with sample size ranging from n=102 to n=144 to n=201. Proportions are derived from N=500 simulated trials, using HS prior distribution and Hill equation parameters corresponding to those in **Supplemental Table 1**. Darker green color represents a higher probability of this ‘good’ event; darker red represents a lower probability of this ‘good’ event. **Abbreviations:** EA, equal allocation; Naïve, naïve allocation; Gr, Greedy allocation; TRInd, targeted randomization (individual); TRCoh, targeted randomization (cohort, comprising 1/3 of study population).

| **Sample Size** | **Allocation Schema** | **Curve 1** | **Curve 2** | **Curve 3** | **Curve 4** | **Curve 5** | **Curve 6** | **Curve 7** | **Curve 8** |
| --- | --- | --- | --- | --- | --- | --- | --- | --- | --- |
|  | **EA** | 17% | 17% | 17% | 17% | 17% | 17% | 17% | 17% |
|  | **Naïve** | 0% | 0% | 0% | 0% | 0% | 63% | 56% | 90% |
| **102** | **Gr** | 46% | 18% | 39% | 65% | 67% | 56% | 83% | 36% |
|  | **TRInd** | 58% | 28% | 45% | 53% | 56% | 50% | 74% | 68% |
|  | **TRCoh*** | 20% | 16% | 25% | 42% | 47% | 39% | 61% | 59% |
|  |  |  |  |  |  |  |  |  |  |
|  | **EA** | 17% | 17% | 17% | 17% | 17% | 17% | 17% | 17% |
|  | **Naïve** | 0% | 0% | 0% | 0% | 0% | 64% | 57% | 92% |
| **144** | **Gr** | 50% | 17% | 42% | 66% | 70% | 59% | 88% | 44% |
|  | **TRInd** | 66% | 31% | 46% | 53% | 58% | 49% | 78% | 68% |
|  | **TRCoh*** | 22% | 18% | 27% | 44% | 49% | 41% | 67% | 63% |
|  |  |  |  |  |  |  |  |  |  |
|  | **EA** | 17% | 17% | 17% | 17% | 17% | 17% | 17% | 17% |
|  | **Naïve** | 0% | 0% | 0% | 0% | 0% | 64% | 57% | 92% |
| **201** | **Gr** | 49% | 16% | 41% | 67% | 70% | 63% | 89% | 45% |
|  | **TRInd** | 72% | 34% | 50% | 57% | 64% | 54% | 82% | 75% |
|  | **TRCoh*** | 24% | 18% | 29% | 45% | 51% | 43% | 70% | 66% |

**Supplemental Table 7. Sensitivity of probability of individual assignment to true MDSE to changes in upper asymptote (estimated maximal efficacy).** Cells contain the average percentage of subjects that were assigned to true MDSE for a given combination of dose allocation strategy and dose-response curve across N=500 simulated trials. Proportions are derived from N=500 simulated trials, using HS prior distribution, n=102, and Hill equation parameters corresponding to those in **Supplemental Table 1**. The upper asymptote of efficacy ranges from 0.85 (curves 1-8) to 0.40 (curves 25-32). Darker green color represents a higher probability of this ‘good’ event; darker red represents a lower probability of this ‘good’ event. **Abbreviations:** EA, equal allocation; Naïve, Naïve allocation; Gr, Greedy allocation; TRInd, targeted randomization (individual); TRCoh, targeted randomization (cohort, comprising 1/3 of study population).

| **Allocation Schema** | **Curve 1** | **Curve 2** | **Curve 3** | **Curve 4** | **Curve 5** | **Curve 6** | **Curve 7** | **Curve 8** |
| --- | --- | --- | --- | --- | --- | --- | --- | --- |
| **EA** | 17% | 17% | 17% | 17% | 17% | 17% | 17% | 17% |
| **Naïve** | 0% | 0% | 0% | 0% | 0% | 63% | 56% | 90% |
| **Gr** | 46% | 18% | 39% | 65% | 67% | 56% | 83% | 36% |
| **TRInd** | 58% | 28% | 45% | 53% | 56% | 50% | 74% | 68% |
| **TRCoh*** | 20% | 16% | 25% | 42% | 47% | 39% | 61% | 59% |
|  | **Curve 9** | **Curve 10** | **Curve 11** | **Curve 12** | **Curve 13** | **Curve 14** | **Curve 15** | **Curve 16** |
| **EA** | 17% | 17% | 17% | 17% | 17% | 17% | 17% | 17% |
| **Naïve** | 0% | 0% | 0% | 0% | 0% | 52% | 47% | 88% |
| **Gr** | 8% | 3% | 14% | 49% | 46% | 62% | 82% | 20% |
| **TRInd** | 27% | 18% | 29% | 43% | 46% | 46% | 64% | 55% |
| **TRCoh*** | 11% | 9% | 19% | 40% | 42% | 37% | 54% | 52% |
|  | **Curve 17** | **Curve 18** | **Curve 19** | **Curve 20** | **Curve 21** | **Curve 22** | **Curve 23** | **Curve 24** |
| **EA** | 17% | 17% | 17% | 17% | 17% | 17% | 17% | 17% |
| **Naïve** | 0% | 0% | 0% | 0% | 0% | 41% | 40% | 85% |
| **Gr** | 2% | 1% | 5% | 47% | 42% | 63% | 81% | 11% |
| **TRInd** | 15% | 11% | 24% | 37% | 39% | 44% | 56% | 45% |
| **TRCoh*** | 7% | 6% | 15% | 38% | 39% | 35% | 47% | 45% |
|  | **Curve 25** | **Curve 26** | **Curve 27** | **Curve 28** | **Curve 29** | **Curve 30** | **Curve 31** | **Curve 32** |
| **EA** | 17% | 17% | 17% | 17% | 17% | 17% | 17% | 17% |
| **Naïve** | 0% | 0% | 0% | 0% | 0% | 35% | 36% | 80% |
| **Gr** | 0% | 0% | 1% | 35% | 32% | 72% | 78% | 5% |
| **TRInd** | 7% | 6% | 17% | 36% | 35% | 41% | 49% | 33% |
| **TRCoh*** | 4% | 4% | 13% | 38% | 37% | 34% | 41% | 36% |

**Supplemental Table 8. Sensitivity of probability of individual assignment to true MDSE to changes in the prior distribution (HS or HSPL).**

Cells contain the average percentage of subjects that were assigned to true MDSE for a given combination of dose allocation strategy and dose-response curve across N=500 simulated trials. Proportions are derived from N=500 simulated trials, using n=102 and Hill equation parameters corresponding to those in **Supplemental Table 1**. Darker green color represents a higher probability of this ‘good’ event; darker red represents a lower probability of this ‘good’ event. **Abbreviations:** EA, equal allocation; Naïve, Naïve allocation; Gr, Greedy allocation; TRInd, targeted randomization (individual); TRCoh, targeted randomization (cohort, comprising 1/3 of study population).

| **Prior Distribution** | **Allocation Schema** | **Curve 1** | **Curve 2** | **Curve 3** | **Curve 4** | **Curve 5** | **Curve 6** | **Curve 7** | **Curve 8** |
| --- | --- | --- | --- | --- | --- | --- | --- | --- | --- |
|  | **EA** | 17% | 17% | 17% | 17% | 17% | 17% | 17% | 17% |
|  | **Naïve** | 0% | 0% | 0% | 0% | 0% | 63% | 56% | 90% |
| **HS** | **Gr** | 46% | 18% | 39% | 65% | 67% | 56% | 83% | 36% |
|  | **TRInd** | 58% | 28% | 45% | 53% | 56% | 50% | 74% | 68% |
|  | **TRCoh*** | 20% | 16% | 25% | 42% | 47% | 39% | 61% | 59% |
|  |  |  |  |  |  |  |  |  |  |
|  | **EA** | 17% | 17% | 17% | 17% | 17% | 17% | 17% | 17% |
|  | **Naïve** | 0% | 0% | 0% | 0% | 0% | 56% | 40% | 87% |
| **HSPL** | **Gr** | 48% | 21% | 43% | 54% | 62% | 52% | 78% | 39% |
|  | **TRInd** | 66% | 38% | 46% | 43% | 53% | 43% | 69% | 65% |
|  | **TRCoh*** | 23% | 20% | 26% | 38% | 44% | 36% | 59% | 57% |

**Supplemental Table 9. Sensitivity of MDSE underestimation to changes in sample size.**

Cells contain the contain the percentage of simulated trials that estimated an MDSE that is *lower* than the true MDSE for a given combination of dose allocation strategy and dose-response curve across N=500 simulated trials, with sample size ranging from n=102 to n=144 to n=201. Proportions are derived from N=500 simulated trials, using HS prior distribution and Hill equation parameters corresponding to those in **Supplemental Table 1**. Darker green color represents a lower probability of this ‘bad’ event; darker red represents a higher probability of this ‘bad’ event. **Abbreviations:** EA, equal allocation; Naïve, Naïve allocation; Gr, Greedy allocation; TRInd, targeted randomization (individual); TRCoh, targeted randomization (cohort, comprising 1/3 of study population).

| **Sample Size** | **Allocation Schema** | **Curve 1** | **Curve 2** | **Curve 3** | **Curve 4** | **Curve 5** | **Curve 6** | **Curve 7** | **Curve 8** |
| --- | --- | --- | --- | --- | --- | --- | --- | --- | --- |
|  | **EA** | 0% | 0% | 2% | 25% | 5% | 37% | 1% | 16% |
|  | **Naïve** | 0% | 0% | 0% | 0% | 0% | 0% | 0% | 6% |
| **102** | **Gr** | 0% | 0% | 0% | 3% | 0% | 16% | 0% | 19% |
|  | **TRInd** | 0% | 0% | 0% | 8% | 0% | 23% | 0% | 10% |
|  | **TRCoh*** | 1% | 0% | 2% | 19% | 3% | 27% | 0% | 7% |
|  |  |  |  |  |  |  |  |  |  |
|  | **EA** | 0% | 0% | 1% | 30% | 3% | 39% | 1% | 11% |
|  | **Naïve** | 0% | 0% | 0% | 0% | 0% | 0% | 0% | 4% |
| **144** | **Gr** | 0% | 0% | 0% | 4% | 0% | 12% | 0% | 14% |
|  | **TRInd** | 0% | 0% | 0% | 12% | 0% | 27% | 0% | 14% |
|  | **TRCoh*** | 0% | 0% | 1% | 20% | 2% | 28% | 0% | 3% |
|  |  |  |  |  |  |  |  |  |  |
|  | **EA** | 0% | 0% | 0% | 27% | 1% | 40% | 0% | 8% |
|  | **Naïve** | 0% | 0% | 0% | 0% | 0% | 0% | 0% | 5% |
| **201** | **Gr** | 0% | 0% | 0% | 2% | 0% | 15% | 0% | 16% |
|  | **TRInd** | 0% | 0% | 0% | 10% | 0% | 26% | 0% | 9% |
|  | **TRCoh*** | 0% | 0% | 0% | 16% | 1% | 24% | 0% | 1% |

**Supplemental Table 10. Sensitivity of MDSE underestimation to changes in upper asymptote (estimated maximal efficacy).** Cells contain the contain the percentage of simulated trials that estimated an MDSE that is *lower* than the true MDSE for a given combination of dose allocation strategy and dose-response curve across N=500 simulated trials. Proportions are derived from N=500 simulated trials, using HS prior distribution, n=102, and Hill equation parameters corresponding to those in **Supplemental Table 1**. The upper asymptote of efficacy ranges from 0.85 (curves 1-8) to 0.40 (curves 25-32). Darker green color represents a lower probability of this ‘bad’ event; darker red represents a higher probability of this ‘bad’ event. **Abbreviations:** EA, equal allocation; Naïve, Naïve allocation; Gr, Greedy allocation; TRInd, targeted randomization (individual); TRCoh, targeted randomization (cohort, comprising 1/3 of study population).

| **Allocation Schema** | **Curve 1** | **Curve 2** | **Curve 3** | **Curve 4** | **Curve 5** | **Curve 6** | **Curve 7** | **Curve 8** |
| --- | --- | --- | --- | --- | --- | --- | --- | --- |
| **EA** | 0% | 0% | 2% | 25% | 5% | 37% | 1% | 16% |
| **Naïve** | 0% | 0% | 0% | 0% | 0% | 0% | 0% | 6% |
| **Gr** | 0% | 0% | 0% | 3% | 0% | 16% | 0% | 19% |
| **TRInd** | 0% | 0% | 0% | 8% | 0% | 23% | 0% | 10% |
| **TRCoh*** | 1% | 0% | 2% | 19% | 3% | 27% | 0% | 7% |
|  | **Curve 9** | **Curve 10** | **Curve 11** | **Curve 12** | **Curve 13** | **Curve 14** | **Curve 15** | **Curve 16** |
| **EA** | 0% | 0% | 2% | 19% | 5% | 30% | 3% | 21% |
| **Naïve** | 0% | 0% | 0% | 0% | 0% | 0% | 0% | 8% |
| **Gr** | 0% | 0% | 0% | 1% | 0% | 7% | 0% | 12% |
| **TRInd** | 0% | 0% | 1% | 14% | 1% | 30% | 2% | 22% |
| **TRCoh*** | 0% | 0% | 1% | 18% | 4% | 30% | 1% | 13% |
|  | **Curve 17** | **Curve 18** | **Curve 19** | **Curve 20** | **Curve 21** | **Curve 22** | **Curve 23** | **Curve 24** |
| **EA** | 0% | 0% | 1% | 13% | 5% | 29% | 7% | 25% |
| **Naïve** | 0% | 0% | 0% | 0% | 0% | 0% | 0% | 12% |
| **Gr** | 0% | 0% | 0% | 3% | 0% | 1% | 0% | 2% |
| **TRInd** | 0% | 0% | 1% | 12% | 3% | 28% | 4% | 32% |
| **TRCoh*** | 0% | 0% | 1% | 12% | 4% | 34% | 3% | 25% |
|  | **Curve 25** | **Curve 26** | **Curve 27** | **Curve 28** | **Curve 29** | **Curve 30** | **Curve 31** | **Curve 32** |
| **EA** | 2% | 2% | 3% | 12% | 8% | 28% | 13% | 41% |
| **Naïve** | 0% | 0% | 0% | 0% | 0% | 0% | 0% | 18% |
| **Gr** | 0% | 0% | 0% | 0% | 0% | 1% | 0% | 0% |
| **TRInd** | 0% | 0% | 0% | 10% | 4% | 26% | 9% | 47% |
| **TRCoh*** | 0% | 0% | 0% | 8% | 3% | 35% | 10% | 39% |

**Supplemental Table 11. Sensitivity of MDSE underestimation to changes in the prior distribution (HS or HSPL).** Cells contain the contain the percentage of simulated trials that estimated an MDSE that is *lower* than the true MDSE for a given combination of dose allocation strategy and dose-response curve across N=500 simulated trials. Proportions are derived from N=500 simulated trials, using n=102 and Hill equation parameters corresponding to those in **Supplemental Table 1**. Darker green color represents a lower probability of this ‘bad’ event; darker red represents a higher probability of this ‘bad’ event. **Abbreviations:** EA, equal allocation; Naïve, Naïve allocation; Gr, Greedy allocation; TRInd, targeted randomization (individual); TRCoh, targeted randomization (cohort, comprising 1/3 of study population).

| **Prior Distribution** | **Allocation Schema** | **Curve 1** | **Curve 2** | **Curve 3** | **Curve 4** | **Curve 5** | **Curve 6** | **Curve 7** | **Curve 8** |
| --- | --- | --- | --- | --- | --- | --- | --- | --- | --- |
|  | **EA** | 0% | 0% | 2% | 25% | 5% | 37% | 1% | 16% |
|  | **Naïve** | 0% | 0% | 0% | 0% | 0% | 0% | 0% | 6% |
| **HS** | **Gr** | 0% | 0% | 0% | 3% | 0% | 16% | 0% | 19% |
|  | **TRInd** | 0% | 0% | 0% | 8% | 0% | 23% | 0% | 10% |
|  | **TRCoh*** | 1% | 0% | 2% | 19% | 3% | 27% | 0% | 7% |
|  |  |  |  |  |  |  |  |  |  |
|  | **EA** | 0% | 0% | 4% | 36% | 8% | 46% | 3% | 20% |
|  | **Naïve** | 0% | 0% | 0% | 0% | 0% | 0% | 0% | 10% |
| **HSPL** | **Gr** | 0% | 0% | 0% | 13% | 1% | 23% | 1% | 30% |
|  | **TRInd** | 0% | 0% | 1% | 25% | 3% | 36% | 1% | 16% |
|  | **TRCoh*** | 1% | 1% | 5% | 31% | 6% | 37% | 0% | 9% |

**Supplemental Table 12. Root-mean-squared error (RMSE) of estimated response rate of estimated MDSE.** Cells contain the RMSE of the estimated MDSE’s efficacy for a given combination of dose allocation strategy and dose-response curve across N=500 simulated trials. Estimates are derived from N=500 simulated trials, using HS prior distribution, n=102 and Hill equation parameters corresponding to those in **Supplemental Table 1**. Darker green color represents lower error; darker red represents a higher error. **Abbreviations:** EA, equal allocation; Naïve, Naïve allocation; Gr, Greedy allocation; TRInd, targeted randomization (individual); TRCoh, targeted randomization (cohort, comprising 1/3 of study population).

| **Allocation Schema** | **Curve 1** | **Curve 2** | **Curve 3** | **Curve 4** | **Curve 5** | **Curve 6** | **Curve 7** | **Curve 8** | **Mean RMSE** |
| --- | --- | --- | --- | --- | --- | --- | --- | --- | --- |
| **EA** | 0.081 | 0.06 | 0.064 | 0.069 | 0.07 | 0.071 | 0.076 | 0.098 | 0.074 |
| **Naïve** | 0.037 | 0.037 | 0.037 | 0.037 | 0.037 | 0.038 | 0.04 | 0.041 | 0.038 |
| **Gr** | 0.037 | 0.038 | 0.038 | 0.04 | 0.038 | 0.044 | 0.076 | 0.12 | 0.054 |
| **TRInd** | 0.042 | 0.041 | 0.041 | 0.046 | 0.047 | 0.051 | 0.051 | 0.048 | 0.046 |
| **TRCoh*** | 0.077 | 0.057 | 0.055 | 0.056 | 0.055 | 0.057 | 0.05 | 0.052 | 0.057 |

**Supplemental Table 13. Sensitivity of RMSE of efficacy estimate to changes in sample size.** Cells contain the RMSE of the estimated MDSE’s efficacy for a given combination of dose allocation strategy and dose-response curve across N=500 simulated trials, with sample size ranging from n=102 to n=144 to n=201. Estimates are derived from N=500 simulated trials, using HS prior distribution and Hill equation parameters corresponding to those in **Supplemental Table 1**. Darker green color represents lower error; darker red represents a higher error. **Abbreviations:** EA, equal allocation; Naïve, Naïve allocation; Gr, Greedy allocation; TRInd, targeted randomization (individual); TRCoh, targeted randomization (cohort, comprising 1/3 of study population).

| **Sample Size** | **Allocation Schema** | **Curve 1** | **Curve 2** | **Curve 3** | **Curve 4** | **Curve 5** | **Curve 6** | **Curve 7** | **Curve 8** | **Mean RMSE** |
| --- | --- | --- | --- | --- | --- | --- | --- | --- | --- | --- |
|  | **EA** | 0.081 | 0.06 | 0.064 | 0.069 | 0.07 | 0.071 | 0.076 | 0.098 | 0.074 |
|  | **Naïve** | 0.037 | 0.037 | 0.037 | 0.037 | 0.037 | 0.038 | 0.04 | 0.041 | 0.038 |
| **102** | **Gr** | 0.037 | 0.038 | 0.038 | 0.04 | 0.038 | 0.044 | 0.076 | 0.12 | 0.054 |
|  | **TRInd** | 0.042 | 0.041 | 0.041 | 0.046 | 0.047 | 0.051 | 0.051 | 0.048 | 0.046 |
|  | **TRCoh*** | 0.077 | 0.057 | 0.055 | 0.056 | 0.055 | 0.057 | 0.05 | 0.052 | 0.057 |
|  |  |  |  |  |  |  |  |  |  |  |
|  | **EA** | 0.068 | 0.052 | 0.052 | 0.063 | 0.06 | 0.066 | 0.069 | 0.084 | 0.064 |
|  | **Naïve** | 0.031 | 0.031 | 0.031 | 0.032 | 0.032 | 0.032 | 0.033 | 0.033 | 0.032 |
| **144** | **Gr** | 0.031 | 0.034 | 0.036 | 0.036 | 0.033 | 0.042 | 0.06 | 0.113 | 0.048 |
|  | **TRInd** | 0.036 | 0.038 | 0.038 | 0.04 | 0.039 | 0.044 | 0.042 | 0.038 | 0.039 |
|  | **TRCoh*** | 0.059 | 0.05 | 0.047 | 0.05 | 0.05 | 0.05 | 0.044 | 0.044 | 0.049 |
|  |  |  |  |  |  |  |  |  |  |  |
|  | **EA** | 0.062 | 0.049 | 0.047 | 0.059 | 0.05 | 0.061 | 0.058 | 0.07 | 0.057 |
|  | **Naïve** | 0.026 | 0.026 | 0.027 | 0.027 | 0.027 | 0.027 | 0.027 | 0.029 | 0.027 |
| **201** | **Gr** | 0.027 | 0.03 | 0.028 | 0.034 | 0.026 | 0.037 | 0.062 | 0.108 | 0.044 |
|  | **TRInd** | 0.028 | 0.034 | 0.031 | 0.037 | 0.035 | 0.036 | 0.032 | 0.031 | 0.033 |
|  | **TRCoh*** | 0.053 | 0.043 | 0.036 | 0.043 | 0.036 | 0.04 | 0.033 | 0.035 | 0.040 |

**Supplemental Table 14. Sensitivity of RMSE of efficacy estimate to changes in upper asymptote (estimated maximal efficacy).** Cells contain the RMSE of the estimated MDSE’s efficacy for a given combination of dose allocation strategy and dose-response curve across N=500 simulated trials. Estimates are derived from N=500 simulated trials, using HS prior distribution, n=102, and Hill equation parameters corresponding to those in **Supplemental Table 1**. The upper asymptote of efficacy ranges from 0.85 (curves 1-8) to 0.40 (curves 25-32). Darker green color represents lower error; darker red represents a higher error. **Abbreviations:** EA, equal allocation; Naïve, Naïve allocation; Gr, Greedy allocation; TRInd, targeted randomization (individual); TRCoh, targeted randomization (cohort, comprising 1/3 of study population).

| **Allocation Schema** | **Curve 1** | **Curve 2** | **Curve 3** | **Curve 4** | **Curve 5** | **Curve 6** | **Curve 7** | **Curve 8** | **Mean RMSE** |
| --- | --- | --- | --- | --- | --- | --- | --- | --- | --- |
| **EA** | 0.081 | 0.06 | 0.064 | 0.069 | 0.07 | 0.071 | 0.076 | 0.098 | 0.074 |
| **Naïve** | 0.037 | 0.037 | 0.037 | 0.037 | 0.037 | 0.038 | 0.04 | 0.041 | 0.038 |
| **Gr** | 0.037 | 0.038 | 0.038 | 0.04 | 0.038 | 0.044 | 0.076 | 0.12 | 0.054 |
| **TRInd** | 0.042 | 0.041 | 0.041 | 0.046 | 0.047 | 0.051 | 0.051 | 0.048 | 0.046 |
| **TRCoh*** | 0.077 | 0.057 | 0.055 | 0.056 | 0.055 | 0.057 | 0.05 | 0.052 | 0.057 |
|  | **Curve 9** | **Curve 10** | **Curve 11** | **Curve 12** | **Curve 13** | **Curve 14** | **Curve 15** | **Curve 16** |  |
| **EA** | 0.08 | 0.066 | 0.069 | 0.071 | 0.076 | 0.081 | 0.089 | 0.108 | 0.080 |
| **Naïve** | 0.044 | 0.046 | 0.044 | 0.045 | 0.044 | 0.045 | 0.046 | 0.046 | 0.045 |
| **Gr** | 0.103 | 0.103 | 0.103 | 0.103 | 0.104 | 0.105 | 0.109 | 0.116 | 0.106 |
| **TRInd** | 0.067 | 0.054 | 0.06 | 0.061 | 0.062 | 0.059 | 0.06 | 0.062 | 0.061 |
| **TRCoh*** | 0.068 | 0.061 | 0.06 | 0.062 | 0.062 | 0.059 | 0.06 | 0.062 | 0.062 |
|  | **Curve 17** | **Curve 18** | **Curve 19** | **Curve 20** | **Curve 21** | **Curve 22** | **Curve 23** | **Curve 24** |  |
| **EA** | 0.084 | 0.08 | 0.078 | 0.08 | 0.082 | 0.084 | 0.091 | 0.103 | 0.085 |
| **Naïve** | 0.05 | 0.05 | 0.05 | 0.049 | 0.05 | 0.049 | 0.049 | 0.05 | 0.050 |
| **Gr** | 0.128 | 0.127 | 0.126 | 0.123 | 0.123 | 0.114 | 0.1 | 0.074 | 0.114 |
| **TRInd** | 0.067 | 0.061 | 0.063 | 0.063 | 0.063 | 0.061 | 0.061 | 0.063 | 0.063 |
| **TRCoh*** | 0.065 | 0.062 | 0.059 | 0.063 | 0.064 | 0.062 | 0.064 | 0.064 | 0.063 |
|  | **Curve 25** | **Curve 26** | **Curve 27** | **Curve 28** | **Curve 29** | **Curve 30** | **Curve 31** | **Curve 32** |  |
| **EA** | 0.082 | 0.079 | 0.077 | 0.08 | 0.079 | 0.081 | 0.084 | 0.091 | 0.082 |
| **Naïve** | 0.049 | 0.048 | 0.049 | 0.048 | 0.048 | 0.048 | 0.048 | 0.048 | 0.048 |
| **Gr** | 0.16 | 0.159 | 0.156 | 0.154 | 0.149 | 0.14 | 0.126 | 0.067 | 0.139 |
| **TRInd** | 0.061 | 0.059 | 0.062 | 0.057 | 0.057 | 0.057 | 0.056 | 0.056 | 0.058 |
| **TRCoh*** | 0.058 | 0.058 | 0.056 | 0.056 | 0.057 | 0.057 | 0.056 | 0.058 | 0.057 |

**Supplemental Table 15. Sensitivity of RMSE of efficacy estimate to changes in the prior distribution (HS or HSPL).** Cells contain the RMSE of the estimated MDSE’s efficacy for a given combination of dose allocation strategy and dose-response curve across N=500 simulated trials. Estimates are derived from N=500 simulated trials, using n=102 and Hill equation parameters corresponding to those in **Supplemental Table 1**. Darker green color represents lower error; darker red represents a higher error. **Abbreviations:** EA, equal allocation; Naïve, Naïve allocation; Gr, Greedy allocation; TRInd, targeted randomization (individual); TRCoh, targeted randomization (cohort, comprising 1/3 of study population).

| **Prior Distribution** | **Allocation Schema** | **Curve 1** | **Curve 2** | **Curve 3** | **Curve 4** | **Curve 5** | **Curve 6** | **Curve 7** | **Curve 8** | **Mean RMSE** |
| --- | --- | --- | --- | --- | --- | --- | --- | --- | --- | --- |
|  | **EA** | 0.081 | 0.06 | 0.064 | 0.069 | 0.07 | 0.071 | 0.076 | 0.098 | 0.074 |
|  | **Naïve** | 0.037 | 0.037 | 0.037 | 0.037 | 0.037 | 0.038 | 0.04 | 0.041 | 0.038 |
| **HS** | **Gr** | 0.037 | 0.038 | 0.038 | 0.04 | 0.038 | 0.044 | 0.076 | 0.12 | 0.054 |
|  | **TRInd** | 0.042 | 0.041 | 0.041 | 0.046 | 0.047 | 0.051 | 0.051 | 0.048 | 0.046 |
|  | **TRCoh*** | 0.077 | 0.057 | 0.055 | 0.056 | 0.055 | 0.057 | 0.05 | 0.052 | 0.057 |
|  |  |  |  |  |  |  |  |  |  |  |
|  | **EA** | 0.073 | 0.058 | 0.072 | 0.072 | 0.079 | 0.075 | 0.081 | 0.103 | 0.077 |
|  | **Naïve** | 0.035 | 0.036 | 0.037 | 0.036 | 0.036 | 0.037 | 0.038 | 0.045 | 0.038 |
| **HSPL** | **Gr** | 0.035 | 0.038 | 0.041 | 0.04 | 0.051 | 0.042 | 0.106 | 0.126 | 0.060 |
|  | **TRInd** | 0.041 | 0.045 | 0.05 | 0.05 | 0.053 | 0.051 | 0.052 | 0.05 | 0.049 |
|  | **TRCoh*** | 0.077 | 0.07 | 0.071 | 0.064 | 0.066 | 0.059 | 0.056 | 0.055 | 0.065 |

**Supplemental Table 16. Sensitivity of administering to an individual subject in the trial a dose less than the true MDSE (underdosing) to changes in sample size.** Cells contain the average percentage of subjects that were assigned to a dose *lower* than true MDSE for a given combination of dose allocation strategy and dose-response curve across N=500 simulated trials, with sample size ranging from n=102 to n=144 to n=201. Proportions are derived from N=500 simulated trials, using HS prior distribution, and Hill equation parameters corresponding to those in **Supplemental Table 1**. Darker green color represents a lower probability of this ‘bad’ event; darker red represents a higher probability of this ‘bad’ event. **Abbreviations:** EA, equal allocation; Naïve, Naïve allocation; Gr, Greedy allocation; TRInd, targeted randomization (individual); TRCoh, targeted randomization (cohort, comprising 1/3 of study population).

| **Sample Size** | **Allocation Schema** | **Curve 1** | **Curve 2** | **Curve 3** | **Curve 4** | **Curve 5** | **Curve 6** | **Curve 7** | **Curve 8** |
| --- | --- | --- | --- | --- | --- | --- | --- | --- | --- |
|  | **EA** | 17% | 17% | 33% | 50% | 50% | 67% | 67% | 83% |
|  | **Naïve** | 0% | 0% | 0% | 0% | 0% | 0% | 0% | 10% |
| **102** | **Gr** | 1% | 1% | 2% | 15% | 5% | 41% | 13% | 64% |
|  | **TRInd** | 4% | 3% | 9% | 27% | 15% | 43% | 15% | 32% |
|  | **TRCoh*** | 6% | 4% | 10% | 27% | 17% | 46% | 20% | 41% |
|  |  |  |  |  |  |  |  |  |  |
|  | **EA** | 17% | 17% | 33% | 50% | 50% | 67% | 67% | 83% |
|  | **Naïve** | 0% | 0% | 0% | 0% | 0% | 0% | 0% | 8% |
| **144** | **Gr** | 0% | 0% | 2% | 15% | 3% | 39% | 8% | 56% |
|  | **TRInd** | 3% | 2% | 8% | 29% | 13% | 45% | 13% | 32% |
|  | **TRCoh*** | 7% | 5% | 10% | 27% | 18% | 46% | 17% | 37% |
|  |  |  |  |  |  |  |  |  |  |
|  | **EA** | 17% | 17% | 33% | 50% | 50% | 67% | 67% | 83% |
|  | **Naïve** | 0% | 0% | 0% | 0% | 0% | 0% | 0% | 8% |
| **201** | **Gr** | 0% | 0% | 1% | 14% | 2% | 35% | 8% | 55% |
|  | **TRInd** | 3% | 2% | 7% | 28% | 12% | 42% | 11% | 25% |
|  | **TRCoh*** | 8% | 5% | 10% | 27% | 17% | 44% | 15% | 34% |

**Supplemental Table 17. Sensitivity of administering to an individual subject in the trial a dose less than the true MDSE (underdosing) to changes in upper asymptote (estimated maximal efficacy).** Cells contain the average percentage of subjects that were assigned to a dose *lower* than true MDSE for a given combination of dose allocation strategy and dose-response curve across N=500 simulated trials. Proportions are derived from N=500 simulated trials, using HS prior distribution and Hill equation parameters corresponding to those in **Supplemental Table 1**. Of note, the upper asymptote of efficacy ranges from 0.85 (curves 1-8) to 0.40 (curves 25-32). Darker green color represents a lower probability of this ‘bad’ event; darker red represents a higher probability of this ‘bad’ event. **Abbreviations:** EA, equal allocation; Naïve, Naïve allocation; Gr, Greedy allocation; TRInd, targeted randomization (individual); TRCoh, targeted randomization (cohort, comprising 1/3 of study population).

| **Allocation Schema** | **Curve 1** | **Curve 2** | **Curve 3** | **Curve 4** | **Curve 5** | **Curve 6** | **Curve 7** | **Curve 8** |
| --- | --- | --- | --- | --- | --- | --- | --- | --- |
| **EA** | 17% | 17% | 33% | 50% | 50% | 67% | 67% | 83% |
| **Naïve** | 0% | 0% | 0% | 0% | 0% | 0% | 0% | 10% |
| **Gr** | 1% | 1% | 2% | 15% | 5% | 41% | 13% | 64% |
| **TRInd** | 4% | 3% | 9% | 27% | 15% | 43% | 15% | 32% |
| **TRCoh*** | 6% | 4% | 10% | 27% | 17% | 46% | 20% | 41% |
|  | **Curve 9** | **Curve 10** | **Curve 11** | **Curve 12** | **Curve 13** | **Curve 14** | **Curve 15** | **Curve 16** |
| **EA** | 17% | 17% | 33% | 50% | 50% | 67% | 67% | 83% |
| **Naïve** | 0% | 0% | 0% | 0% | 0% | 0% | 0% | 12% |
| **Gr** | 0% | 0% | 1% | 7% | 3% | 35% | 14% | 80% |
| **TRInd** | 3% | 2% | 8% | 26% | 16% | 44% | 21% | 45% |
| **TRCoh*** | 3% | 2% | 7% | 21% | 16% | 44% | 24% | 48% |
|  | **Curve 17** | **Curve 18** | **Curve 19** | **Curve 20** | **Curve 21** | **Curve 22** | **Curve 23** | **Curve 24** |
| **EA** | 17% | 17% | 33% | 50% | 50% | 67% | 67% | 83% |
| **Naïve** | 0% | 0% | 0% | 0% | 0% | 0% | 0% | 15% |
| **Gr** | 0% | 0% | 0% | 3% | 1% | 34% | 16% | 89% |
| **TRInd** | 2% | 2% | 7% | 22% | 15% | 42% | 26% | 55% |
| **TRCoh*** | 1% | 1% | 5% | 17% | 14% | 44% | 29% | 55% |
|  | **Curve 25** | **Curve 26** | **Curve 27** | **Curve 28** | **Curve 29** | **Curve 30** | **Curve 31** | **Curve 32** |
| **EA** | 17% | 17% | 33% | 50% | 50% | 67% | 67% | 83% |
| **Naïve** | 0% | 0% | 0% | 0% | 0% | 0% | 0% | 20% |
| **Gr** | 0% | 0% | 0% | 1% | 0% | 25% | 20% | 95% |
| **TRInd** | 1% | 1% | 4% | 19% | 16% | 43% | 33% | 67% |
| **TRCoh*** | 0% | 0% | 3% | 14% | 12% | 44% | 35% | 64% |

**Supplemental Table 18. Sensitivity of administering to an individual subject in the trial a dose less than the true MDSE (underdosing) to changes in the prior distribution (HS or HSPL).** Cells contain the average percentage of subjects that were assigned to a dose *lower* than true MDSE for a given combination of dose allocation strategy and dose-response curve across N=500 simulated trials. Proportions are derived from N=500 simulated trials, using n=102 and Hill equation parameters corresponding to those in **Supplemental Table 1**. Darker green color represents a lower probability of this ‘bad’ event; darker red represents a higher probability of this ‘bad’ event. **Abbreviations:** EA, equal allocation; Naïve, Naïve allocation; Gr, Greedy allocation; TRInd, targeted randomization (individual); TRCoh, targeted randomization (cohort, comprising 1/3 of study population).

| **Prior Distribution** | **Allocation Schema** | **Curve 1** | **Curve 2** | **Curve 3** | **Curve 4** | **Curve 5** | **Curve 6** | **Curve 7** | **Curve 8** |
| --- | --- | --- | --- | --- | --- | --- | --- | --- | --- |
|  | **EA** | 17% | 17% | 33% | 50% | 50% | 67% | 67% | 83% |
|  | **Naïve** | 0% | 0% | 0% | 0% | 0% | 0% | 0% | 10% |
| **HS** | **Gr** | 1% | 1% | 2% | 15% | 5% | 41% | 13% | 64% |
|  | **TRInd** | 4% | 3% | 9% | 27% | 15% | 43% | 15% | 32% |
|  | **TRCoh*** | 6% | 4% | 10% | 27% | 17% | 46% | 20% | 41% |
|  |  |  |  |  |  |  |  |  |  |
|  | **EA** | 17% | 17% | 33% | 50% | 50% | 67% | 67% | 83% |
|  | **Naïve** | 0% | 0% | 0% | 0% | 0% | 0% | 0% | 13% |
| **HSPL** | **Gr** | 1% | 1% | 3% | 22% | 9% | 44% | 17% | 61% |
|  | **TRInd** | 5% | 4% | 12% | 38% | 20% | 48% | 18% | 35% |
|  | **TRCoh*** | 9% | 6% | 14% | 31% | 21% | 48% | 21% | 43% |
